# Pseudo Primary Aldosteronism as Initial Presentation of Ectopic ACTH Syndrome in Metastatic Small Cell Lung Cancer

**DOI:** 10.1210/jcemcr/luac020

**Published:** 2022-11-30

**Authors:** Jonathan Shakesprere, Reima El Naili, Mehjabeen Sadiq, Adnan Haider

**Affiliations:** Department of Internal Medicine, West Virginia University School of Medicine, Morgantown, WV 26505, USA; Section of Endocrinology and Metabolism, Department of Pathology, Anatomy and Laboratory Medicine, West Virginia University School of Medicine, Morgantown, WV 26505, USA; Department of Internal Medicine, West Virginia University School of Medicine, Morgantown, WV 26505, USA; Department of Internal Medicine, West Virginia University School of Medicine, Morgantown, WV 26505, USA

**Keywords:** hyperaldosteronism, paraneoplastic ACTH secretion, dexamethasone suppression test

## Abstract

Ectopic adrenocorticotropic hormone (ACTH)-secreting syndrome (EAS) is a rare but often aggressive paraneoplastic syndrome. Patients with EAS typically present with high ACTH levels and rapid clinical progression in the setting of acute cortisol elevation, which can delay diagnosis due to a lack of typical Cushingoid features. High levels of ACTH have also been shown to stimulate the adrenal zona glomerulosa to oversecrete aldosterone. We present the case of a 58-year-old male individual presenting with new-onset hypertension and severe metabolic alkalosis with spontaneous hypokalemia, in the setting of elevated aldosterone and low renin levels, suggestive of primary aldosteronism. Subsequent biochemical testing, imaging, and pathology, however, revealed suppression of aldosterone with evidence of hypercortisolism in the setting of metastatic small cell lung cancer. This was, therefore, suggestive of pseudo primary aldosteronism in the setting of a paraneoplastic ectopic ACTH-producing syndrome. This case highlights that hypercortisolism, in the setting of EAS, can initially present with a clinical picture suggestive of hyperaldosteronism. The use of a dexamethasone suppression test can allow the clinician to differentiate between idiopathic bilateral adrenal hyperplasia and ectopic ACTH syndrome.

Ectopic adrenocorticotropic hormone (ACTH)-secreting syndrome (EAS) has been recognized as a rare but often aggressive paraneoplastic syndrome caused by non-pituitary tumors. Patients with EAS typically present with elevated ACTH levels and rapid clinical progression in the setting of acute cortisol elevations, which can cause a delay in diagnosis due to a lack of typical Cushingoid features (1). High levels of ACTH have also been shown to stimulate the adrenal zona glomerulosa to overproduce aldosterone, which can present with hypertension, hypokalemia, and acid-base abnormalities (2). However, a review of current literature reporting hyperaldosteronism, characterized by the above findings along with elevated aldosterone and low renin levels, in the setting of an ectopic ACTH syndrome, is limited.

## Case Presentation

A 58-year-old incarcerated Caucasian male without significant past medical history initially presented to an outside facility with a chief complaint of new-onset chest pain. Laboratory workup incidentally revealed severe metabolic alkalosis of pH 7.74 with elevated serum bicarbonate of 33 mmol/L and hypokalemia of 1.8 mmol/L. He was subsequently transferred to our hospital for further management. History obtained from patient was significant for no personal or family history of adrenal disease or Cushing syndrome, as well as the absence of any previous diagnoses of hypertension or electrolyte abnormalities.

## Diagnostic Assessment and Treatment

**Figure 1.**
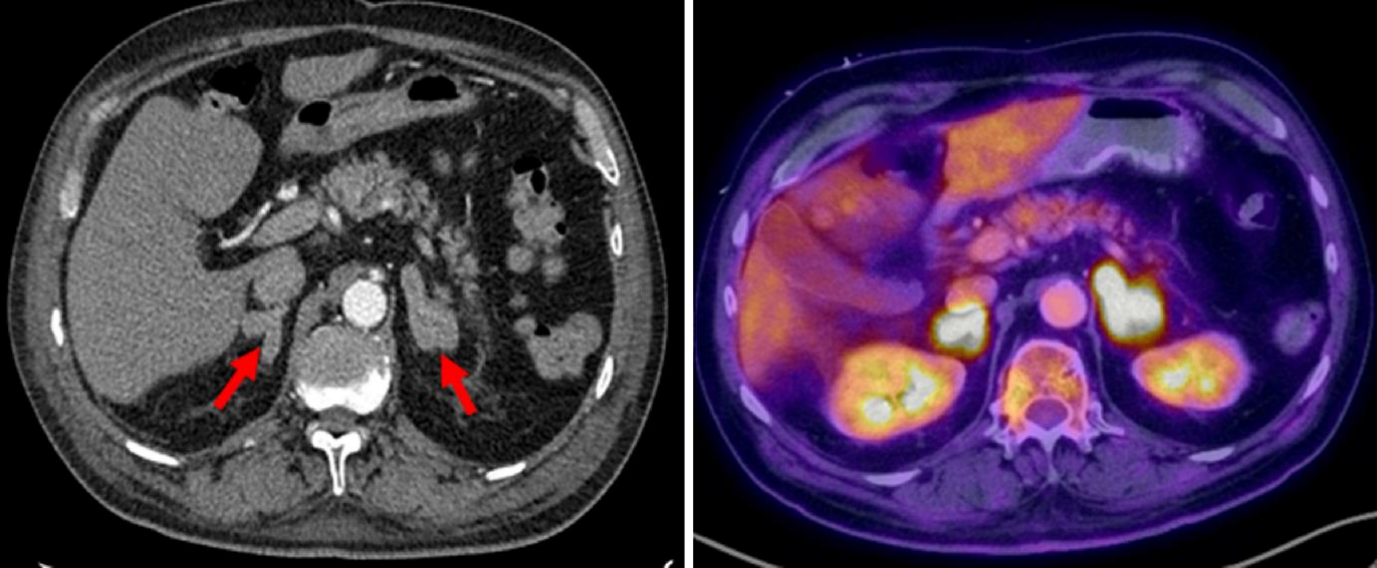
Bilateral adrenal glands thickening without discrete nodules on CT abdomen/pelvis (left) and PET/CT (indicated by red arrows).

Central venous access was placed for intensive potassium repletion due to profound hypokalemia. Aldosterone and renin levels were ordered the day after admission, collected in the supine position, and measured via liquid chromatography/tandem mass spectrometry (LC/MS-MS) assay. CT Adrenal protocol revealed diffuse bilateral adrenal gland thickening without discrete masses (Fig. 1), as well as an incidental pancreatic tail mass. Hounsfield units were not provided as nodularity was not noted. CT Chest was then obtained, revealing a large right hilar lung mass with mediastinal lymphadenopathy suspicious for primary lung malignancy. Subsequent MRI of the abdomen was concerning for metastatic disease with numerous small-rounded lesions scattered throughout the axial skeleton. GI Advanced Endoscopy and Thoracic Surgery services were consulted for biopsies of the pancreatic and mediastinal masses, respectively.

Initial renin and aldosterone testing results returned, revealing elevated serum aldosterone of 110 ng/dL (RR, ≤ 28 ng/dL), low plasma renin activity (PRA) of 0.35 ng/mL/hour (RR, 0.167-5.38 ng/mL/hour), and an elevated aldosterone/PRA ratio of 314.3 (RR, 0.9-28.9 ratio; Table 1) Serum potassium at the time of testing was 1.7 mg/dL. Metanephrines, either urine or serum, were not obtained, as both clinical presentation and adrenal imaging were not consistent with pheochromocytoma. The patient was started on spironolactone 50 mg daily, after initial aldosterone and renin levels returned, for hypertension. Angiotensin converting enzyme inhibitor or angiotensin II receptor blocker therapies were not started, due to reduced creatinine clearance.

**Table 1.** Patient's laboratory investigation: baseline values and cortisol testing results

Parameter	Value	Reference range
Serum sodium	139	137-145 mmol/L
Serum potassium	1.7	3.5-5.1 mmol/L
Serum bicarbonate	45	22-30 mmol/L
Serum glucose	165	74-106 mg/dL
Serum magnesium	2.0	1.6-2.3 mg/dL
Arterial pH	7.74	7.35-7.45
Aldosterone	110	≤28 ng/dL
Plasma renin activity	0.35	0.25-5.82 ng/mL
Aldo/PRA ratio	314.3	0.9-28.9 ratio
ACTH	362.7	7.7-77.0 pg/mL
3 mg DST	118	<1.8 ug/dL
4 mg DST	132.9	<1.8 ug/dL
24-hour free cortisol	28 260.0	3.1-42.3 mcg/g Cr

Abbreviations: ACTH, adrenocorticotropic hormone; DST, dexamethasone suppression test; PRA, plasma renin activity.

Endocrinology was consulted due to concern for primary aldosteronism. No signs of Cushing disease were noted on exam, including facial plethora, central obesity, or proximal muscle weakness. An overnight 3 mg dexamethasone suppression test (DST) was completed showing an unsuppressed morning cortisol level of 118 ug/dL (RR, <1.8 ug/dL); it was noted to be drawn at 4 Am instead of 8 Am. A higher dose dexamethasone test, rather than the standard 1 mg dose, was used to ensure testing adequacy and steroid absorption in the setting of the patient's obesity (body mass index of 30) and prolonged immobility. A morning ACTH level was significantly elevated at 362.7 pg/dL (RR, 7-77 pg/mL). A hemoglobin A1c level was found to be elevated to 7.2% (SI: 55 mmol/mol), marking a new diagnosis of diabetes mellitus.

Ectopic ACTH syndrome was considered as a possible paraneoplastic manifestation of an underlying lung vs neuroendocrine tumor. As initial lab work showed elevated aldosterone with low renin levels rather than suppressed aldosterone and renin levels, a syndrome of apparent mineralocorticoid excess was less likely. Concurrent ACTH-independent adrenal co-secretion of cortisol was ruled out based on elevated ACTH level and non-suppressed morning cortisol on the DST. Idiopathic bilateral adrenal hyperplasia was suggested based on high aldosterone and low renin levels in the setting of bilateral adrenal enlargement. The decision was made to undergo adrenal vein sampling (AVS) to rule out a potential rare coexistence of primary aldosteronism from bilateral idiopathic adrenal hyperplasia and ectopic ACTH syndrome. Prior to AVS completion, low-dose amiloride was started due to persistent hypertension and the patient remained on spironolactone, up-titrated during the hospital course to 150 mg daily.

A repeat dexamethasone suppression test, timed appropriately, still showed unsuppressed morning cortisol at 132.9 ng/dL (Table 1). A serum dexamethasone level was 964 ng/dL (RR, ≥200 ng/dL), indicating that the DST was valid, with appropriate absorption of the dexamethasone tablet. Midnight cortisol level was elevated at 61.9 ng/dL (RR, <2 ng/dL), indicating loss of diurnal circadian rhythm. 24-hour urine free cortisol was severely elevated at 28 260 mcg/g Cr (RR, 3.1-42.3 mcg/g Cr). His dehydroepiandrosterone sulfate level was normal at 174 ug/dL (RR, 70-310 µg/dL). AVS results then returned, notably revealing suppressed aldosterone bilaterally and in the inferior vena cava. This suggested that acutely elevated cortisol levels were likely now causing mineralocorticoid suppression and that previously demonstrated hyperaldosteronism was transient, driven by acutely rising ACTH levels. Of note, right adrenal vein cannulation demonstrated a lack of cortisol gradient between the right adrenal vein and inferior vena cava, possibly related to procedural inconsistency.

## Outcome and Follow-Up

Histopathology from mediastinal and pancreatic mass biopsies was ultimately consistent with high-grade neuroendocrine carcinoma with small cell features. Positron emission tomography/computed tomography (PET/CT) was completed, showing bilateral adrenal hypermetabolic nodular thickening (Fig. 1) as well extensive-stage small cell disease. Medical Oncology was consulted for initiation of inpatient chemotherapy. Initiation of octreotide, as well as ketoconazole, for cortisol-lowering therapy was considered but deferred as the patient had been started on treatment for rapid tumor reduction with anticipated reduction in ACTH production.

**Figure 2.**
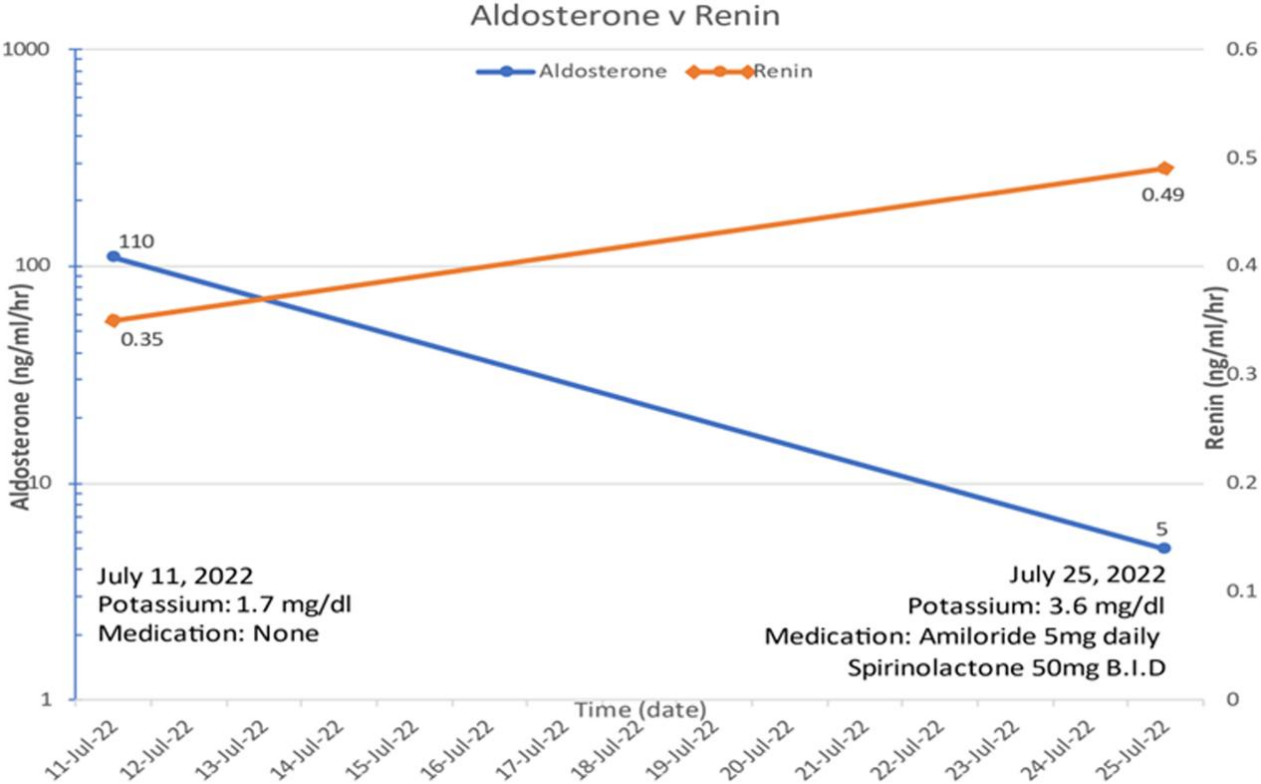
Serum aldosterone vs plasma renin activity levels, from initial testing to repeat testing, during hospital admission. Blue line: Aldosterone level (ng/mL/hour); Orange line: Renin level (ng/mL/hour). Potassium level (mg/dL); inpatient potassium-sparing diuretic medications and doses (mg) listed.

Repeat renin and aldosterone levels returned prior to discharge, now showing a normal aldosterone level of 5 ng/dL, PRA of 0.49 ng/mL/hour, and aldosterone/PRA ratio of 10.2; serum potassium was 3.6 mg/dL at the time of repeat draw (Fig. 2). The patient was discharged after his first cycle of chemotherapy was completed with repeat ACTH and biweekly cortisol levels and close outpatient Endocrinology follow-up. Unfortunately, it was discovered that the patient was unable to tolerate further treatment cycles at his correctional facility and ultimately passed away shortly after discharge prior to follow-up.

## Discussion

Under physiological conditions, blood aldosterone levels are regulated mainly by angiotensin II, serum potassium levels, and to a lesser extent, adrenocorticotropic hormone (ACTH).

ACTH, a pituitary hormone consisting of 39 amino acids, binds its specific receptor, melanocortin 2 receptor, expressed by the adrenocortical cells (3). Upon binding to melanocortin 2 receptor, ACTH acutely activates the intracellular cAMP-protein kinase A pathway, thereby increasing the expression of the steroidogenic acute regulatory protein (StAR), which regulates the production of steroids. In the zona glomerulosa of the adrenal cortex, which is the major source of circulating aldosterone under physiological conditions, upregulated StAR results in an increase in the production of aldosterone (4). ACTH also increases adrenal weight by inducing both hyperplasia and hypertrophy of the adrenal gland, which was noted bilaterally in our patient (Fig. 1).

The effect of ACTH on aldosterone secretion differs in acute and chronic situations. Honour et al published aldosterone responses to different doses of cosyntropin in healthy volunteers and demonstrated an increase in aldosterone levels starting 10 minutes after intravenous cosyntropin. Interestingly, there were no differences in peak aldosterone response to different ACTH doses (5). Whereas a near-maximal cortisol response was attained with a cosyntropin dose of 500 ng/m^2^, aldosterone response was already maximal at 125 ng/m^2^ and, for approximately every 1 pmol/L increase in aldosterone, there was a 1.25 nmol/L rise in cortisol. Another study done by Diadoh and colleagues showed that a 25 mg ACTH dose had the same releasing effect as a 250 mg dose on aldosterone secretion (6). This suggests that the zona glomerulosa responds to extremely low ACTH variations. Their findings also showed that the aldosterone response to ACTH was almost invariably recorded 15 minutes after ACTH administration. In contrast, chronic continual ACTH stimulation has been shown to have either no effect or an inhibitory effect on aldosterone production. It is hypothesized that chronic stimulation causes receptor downregulation or suppression of angiotensin II-stimulated secretion because of a mineralocorticoid effect of cortisol, deoxycorticosterone, and corticosterone (7).

The aforementioned contrast of cortisol and aldosterone responses to ACTH is likely pertinent in our case. We hypothesize that the small cell lung cancer in our patient secreted excess ACTH, to which the zona glomerulosa of the adrenal glands initially responded by making more aldosterone, as his cortisol levels had not peaked yet. Therefore, at time of presentation, initial laboratory work was suggestive of primary aldosteronism. At the time of initial testing, the patient was not on any antihypertensive medications; a falsely elevated aldosterone level was unlikely, especially in the setting of severe hypokalemia. Although laboratory error may be considered as a reason for very high aldosterone levels initially, liquid chromatography–tandem mass spectrometry (LC-MS/MS) testing does not show cross-reactivity with other steroids or their metabolites, unlike antibody-mediated assays which can cross-react with alternative steroid immunoassays. Intravascular volume depletion from untreated hyperglycemia could have initially presented with high aldosterone with high renin levels, but this was not seen in our patient.

During the hospital course, as abdominal imaging revealed no identifiable adrenal mass, ACTH production by the cancer cells acutely increased cortisol production, as evidenced by the very high 24-hour urine free cortisol (Table 1). High circulating cortisol levels likely overwhelmed the cortisol-cortisone shunt, resulting in 11β-hydroxysteroid dehydrogenase type 2 enzymes being unable to convert excess cortisol to inactive cortisone. This resulted in remaining high levels of circulating cortisol exerting a mineralocorticoid effect on the distal intercalated cells of the renal nephron, resulting in low aldosterone, as well as low renin, with significant hypokalemia and metabolic alkalosis (8). This suggested pseudo primary aldosteronism, with initially driven rising ACTH levels and later suppressed, as evidenced on AVS and repeat laboratory workup (Fig. 2).

As noted previously, the right adrenal side was not cannulated properly as shown by the lack of cortisol gradient between the right adrenal vein and inferior vena cava; however, suppressed aldosterone in the inferior vena cava was consistent with the postulated hypothesis of ACTH stimulation causing aldosterone suppression after initial stimulation. Our patient was on spironolactone and amiloride prior to AVS for 10 and 4 days, respectively; however, these medications were unlikely to have changed the results of the AVS. Potassium-sparing diuretics, including spironolactone and amiloride, increase both serum aldosterone and renin levels (9). In our patient, AVS showed suppressed aldosterone and renin on the left side and in the inferior vena cava. Furthermore, repeat laboratory workup prior to discharge showed decreased aldosterone without significant renin elevation while actively taking these medications (Fig. 2).

## Learning Points

- This case highlights that hypercortisolism in the setting of ectopic ACTH secretion can present with acid-base disturbances, electrolyte abnormalities, and hypertension suggestive of hyperaldosteronism.
- Although initial screening tests and exam findings may suggest a more benign etiology, a high index of suspicion to evaluate for underlying malignancy is warranted.
- Use of a dexamethasone suppression test can help the clinician to identify excess endogenous cortisol production as the culprit in the setting of paraneoplastic ACTH secretion.

## Data Availability

Data sharing is not applicable to this article as no datasets were generated or analyzed during the current study.

## Abbreviations

ACTH: adrenocorticotropin hormone
AVS: adrenal vein sampling
DST: dexamethasone suppression test
EAS: ectopic ACTH syndrome
PET/CT: positron emission tomography/computed tomography
PRA: plasma renin activity ratio
StAR: steroidogenic acute regulatory protein
